# Affective symptoms and swallow‐specific quality of life in total laryngectomy patients

**DOI:** 10.1002/hed.26365

**Published:** 2020-07-04

**Authors:** Glen J. F. Kemps, Iris Krebbers, Walmari Pilz, Sophie Vanbelle, Laura W. J. Baijens

**Affiliations:** ^1^ Department of Otorhinolaryngology, Head and Neck Surgery Maastricht University Medical Center Maastricht The Netherlands; ^2^ School for Oncology and Developmental Biology (GROW) Maastricht University Maastricht The Netherlands; ^3^ School of Mental Health and Neurosciences (MHeNS) Maastricht University Maastricht The Netherlands; ^4^ Department of Methodology and Statistics Maastricht University Maastricht The Netherlands; ^5^ Care and Public Health Research Institute – CAPHRI Maastricht University Medical Center Maastricht The Netherlands

**Keywords:** dysphagia, HADS, laryngectomy, MDADI, swallow‐specific quality of life

## Abstract

**Background:**

The aim of this study is to determine the prevalence of clinically relevant affective symptoms and level of swallow‐specific quality of life (QoL) in dysphagic patients with total laryngectomy (TL) and to explore the relationship between affective symptoms and swallow‐specific QoL.

**Methods:**

Thirty‐five TL patients completed the Hospital Anxiety and Depression Scale (HADS) and the MD Anderson Dysphagia Inventory (MDADI). Student's *t* test and linear regression were used.

**Results:**

Eight (23%) patients showed clinically relevant symptoms of anxiety, 8 (23%) of depression, and 11 (31%) showed either one. These groups had significantly lower mean MDADI scores. One‐point increase in HADS‐anxiety or HADS‐depression subscale score corresponds with a decrease of 2.7 or 3.0 points, on average, respectively, of the MDADI total score.

**Conclusions:**

Clinically relevant affective symptoms were present in approximately one‐third of the TL patients. These preliminary results show that increased affective symptom scores correlate with a decreased swallow‐specific QoL.

## 1 INTRODUCTION

Laryngeal cancer is the most common malignancy of the upper aerodigestive tract, and total laryngectomy (TL) is the treatment of choice in advanced tumor stages with thyroid cartilage invasion.^1^, ^2^ Although patients with head and neck cancer (HNC) presenting advanced laryngeal cancer usually receive primary (chemo)radiotherapy, patients with a dysfunctional larynx (airway obstruction, severe oropharyngeal dysphagia [OD], severe dysphonia, etc.) will preferably undergo a primary TL, followed by postoperative radiotherapy if indicated.^3^, ^4^ In cases of recurrent tumor following (chemo)radiotherapy, a salvage TL is necessary, if possible. TL is a major surgical intervention in which the larynx is entirely removed. A TL with pharyngectomy or even cervical esophagectomy and free flap reconstruction or gastric pull up may also be indicated for hypopharyngeal cancer invading the larynx or for laryngeal cancer invading the pharynx.^5^ Following TL, communication, respiration, and swallowing function will drastically change and the prevalence of postoperative OD may not be disregarded.^6^, ^7^ It has been shown that the anatomy and physiology of the newly created pharynx may vary according to the type of surgical reconstruction,^6^ which can therefore affect swallowing. OD following TL and, in particular salvage TL, can be caused by fibrosis of pharyngeal constrictors,^6^, ^8^, ^9^ neopharyngeal strictures,^10^, ^11^ pseudovallecula,^9^ radiotherapy‐induced sensorial neuropathy,^12^ xerostomia,^12^ etc. The main swallowing complaints reported by TL patients are nasopharyngeal regurgitation, food “sticking” in the throat, aerophagia, noisy swallowing, and a prolonged mealtime. These complaints may result in avoidance of participation in social activities, such as having a meal with family or friends.^7^, ^13^

Previous studies have already investigated the impact of a TL on health‐related quality of life (QoL).^13^, ^14^, ^15^, ^16^ However, these studies used health‐related QoL questionnaires that do not measure swallow‐specific QoL.^15^, ^16^ The use of a swallow‐specific questionnaire is paramount to understand the impact of swallowing impairment on the QoL of TL patients. The impact of affective symptoms (depression, anxiety) on social relationship,^14^, ^15^, ^17^ health‐related QoL,^13^, ^14^, ^18^ speech intelligibility,^14^, ^15^, ^17^, ^19^ and quality of the substitution voice^1^ of TL patients has been reported in the literature. To the best of our knowledge, no study has been published on the relationship between affective symptoms and swallow‐specific QoL in TL patients. It is hypothesized that both variables probably have a close relationship with each other and that in the clinical context, attention should be paid to OD management but also to the association between OD and psychosocial well‐being in TL patients. Therefore, the aim of this study was twofold: (a) to determine the prevalence and severity of clinically relevant affective symptoms and the level of swallow‐specific QoL in dysphagic TL patients and (b) to explore the relationship between affective symptoms and swallow‐specific QoL.

## 2 MATERIALS AND METHODS

### 2.1 Participants

For this cross‐sectional observational study, TL patients were recruited from the outpatient clinic for OD of a tertiary referral hospital over a period of 6 years (2013‐2018). In this period 60 patients underwent a TL. Patients were only included in the present study when they presented themselves at the outpatient clinic for OD. Patients were referred to this outpatient clinic by the speech and language pathologist, the radiation oncologist, the head and neck surgeon, or the general practitioner. All included patients presented OD complaints such as prolonged mealtime, residue in the oral cavity after the meal, and nasopharyngeal regurgitation. Patients were enrolled in the study if their malignancy was in remission. The exclusion criteria were: presenting with a concurrent neurological disease (stroke, Parkinson's, etc.), scoring below 23 on a Mini Mental State Examination (MMSE), being older than 85 years, having a second primary head and neck tumor, having osteoradionecrosis of the maxilla or mandible, and being illiterate or blind. None of the patients was in a palliative state of disease. Cancer staging according to the tumor, nodes, and metastasis (T classification, N classification, and M classification) classification system was performed.^20^ Informed consent for the clinical OD protocol was obtained from all patients and the study protocol was approved by the medical ethics committee in compliance with the Medical Research Involving Human Subjects Act (*Wet Medisch‐Wetenschappelijk Onderzoek* [WMO]) as non‐WMO research.^21^ The surgical techniques applied were strictly standardized, including a myotomy of the upper esophageal sphincter, closure of the neopharynx in three layers using the same sutures, and insertion of a tracheoesophageal speech prosthesis in all patients. All patients having an indication for radiotherapy underwent intensity‐modulated radiotherapy.

### 2.2 Swallowing assessment protocol

A standardized swallowing protocol, used in daily clinical practice at the outpatient clinic for OD, was carried out. All patients underwent a clinical ear, nose, and throat examination (including integrity of the remaining cranial nerves) performed by a laryngologist, measurement of body mass index, and the Functional Oral Intake Scale (FOIS).^22^ The FOIS is a dietary intake scale. The range of scores of the FOIS is 1 to 7, where 1 corresponds with “no oral diet, nothing by mouth” and 7 with “total oral diet, no restrictions.” The severity of disabled dietary intake may not be a determinant of the severity of OD. All patients completed the Hospital Anxiety and Depression Scale (HADS)^23^, ^24^ and the MD Anderson Dysphagia Inventory (MDADI) questionnaire.^25^

The HADS is a self‐report questionnaire designed to assess patients' affective symptoms. It was translated to and validated in the Dutch language.^26^ The questionnaire contains 14‐items, 7 related to anxiety (HADS‐A) and 7 related to depression (HADS‐D) symptoms. Each item has a Likert response scale ranging from 0 to 3, where 0 indicates absence of symptoms and 3 continuous presence of symptoms. HADS‐A and HADS‐D scores range from 0 to 21. The sum of these scores composes the HADS total (HADS‐T) score of which the maximum is 42 points. A score of 8 or more on the HADS‐A and/or HADS‐D subscale represents the presence of clinically relevant symptoms of anxiety and/or depression.^23^

The MDADI is a self‐report, psychometrically validated and reliable questionnaire used to assess the impact of OD on the health‐related QoL of patients with HNC.^25^ As in the original version, the validated Dutch translation of the MDADI consists of 20 items divided in 4 subscales: the global (single item), functional (5 items), physical (8 items), and emotional (6 items) subscale.^27^ The global subscale (MDADI‐G) reflects the effect of patients' swallowing ability on overall health‐related QoL. The functional subscale (MDADI‐F) represents the impact of OD on daily activities. The physical subscale (MDADI‐P) refers to patients' self‐perception of his/her swallowing difficulty. The emotional subscale (MDADI‐E) refers to the patients' affective response to the swallowing disorder. All items are rated on a 5‐point scale (1–5) where 1 corresponds with “strongly agree” and 5 to “strongly disagree.” The maximum score for each subscale is 100 points and the minimum score is 20, which is calculated by dividing the subscale score by the amount of questions it entails and multiplying it by 20. The MDADI total score (MDADI‐T) was determined by the sum of the scores of all subscales except for the MDADI‐G, subsequently divided by 19 and then multiplied by 20. A low score indicates low functioning and a high score indicates high functioning.

### 2.3 Statistical analysis

Descriptive statistics were reported as means (SD) for continuous variables and numbers (percentage) for categorical variables. The MDADI and HADS questionnaires were reviewed for possible floor and ceiling effects, noting the number of respondents who obtained the lowest or highest possible scores on these questionnaires. Both the nonparametric Mann‐Whitney U and parametric Student's *t* test were performed to test for differences in the MDADI scores between patients with vs patients without clinically relevant affective symptoms. Only parametric results are reported since nonparametric tests revealed similar results. Linear regression was used to explore the correlation between MDADI scores and HADS‐A/D scores (0‐21 points per subscale). All statistical analyses were performed using IBM SPSS Statistics for Windows, version 23 (Armonk, New York: IBM Corp.). A *P*‐value ≤.05 was considered statistically significant.

## 3 RESULTS

### 3.1 Patient characteristics

Thirty‐five TL patients were enrolled in the analysis of both HADS and MDADI scores. The age ranged from 43 to 82 years with a mean (SD) age of 69 (9). Thirty‐eight patients were included. However, one patient did not complete the HADS questionnaire, and two patients did not complete one of the MDADI subscales, so their MDADI‐T score could not be calculated. Data collection took place at least 4 months after HNC treatment. Patient characteristics are presented in Table 1. The median (interquartile range [IQR]) FOIS score was 6 (5‐6). No statistically significant difference in average FOIS score was found between patients with vs patients without clinically relevant affective symptoms. Five patients (14%) were using psychotropic drugs at the time of the examination, that is, antidepressant and/or anxiolytic drugs. Four (11%) patients underwent adjuvant systemic therapy during multimodality treatment (n = 1 primary bioradiotherapy before salvage TL; n = 3 postoperative concurrent chemoradiotherapy). These subgroups were considered too small for meaningful statistical analysis. None of the patients suffered from cranial nerve impairment other than of the recurrent laryngeal nerve.

**TABLE 1:** Patient characteristics

Gender (No. of patients; %)	Total	HADS‐A≥8	HADS‐A<8	HADS‐D≥8	HADS‐D<8
Male	33 (94%)	7 (21%)	26 (79%)	7 (21%)	26 (79%)
Female	2 (6%)	1 (50%)	1 (50%)	1 (50%)	1 (50%)
Mean age (SD)	69 (9)	68 (8)	69 (9)	68 (9)	69 (9)
Mean BMI (SD)	26.2 (4.5)	25.8 (5.3)	26.3 (4.4)	25.5 (4.1)	26.4 (4.6)
Mean time after treatment in months (SD)	85 (109)	27 (16)	102 (119)	35 (26)	100 (120)
FOIS (no. of patients; %)					
Level ≤4	0 (0%)	0 (0%)	0 (0%)	0 (0%)	0 (0%)
Level 5	13 (37%)	4 (31%)	9 (69%)	4 (31%)	9 (69%)
Level 6	8 (23%)	2 (25%)	6 (75%)	2 (25%)	6 (75%)
Level 7	6 (17%)	0 (0%)	6 (100%)	0 (0%)	6 (100%)
Missing values	8 (23%)	2 (25%)	6 (75%)	2 (25%)	6 (75%)
Total	35 (100%)	8 (23%)	27 (77%)	8 (23%)	27 (77%)
Treatment (no. of patients; %)					
TL (single modality)	2 (6%)	1 (50%)	1 (50%)	1 (50%)	1 (50%)
TL + (chemo)radiotherapy^a^	11 (31%)	3 (27%)	8 (73%)	2 (18%)	9 (82%)
Salvage TL^b^	22 (63%)	4 (18%)	18 (82%)	5 (23%)	17 (77%)
Psychotropic drugs (no. of patients; %)					
Yes	5 (14%)	2 (40%)	3 (60%)	2 (40%)	3 (60%)
No	30 (86%)	6 (20%)	24 (80%)	6 (20%)	24 (80%)
Tracheoesophegeal speech					
Yes	35 (100%)				
No	0 (0%)				

^a^ Concurrent systemic therapy following TL was composed of cisplatin (chemoradiation; no. of patients = 3).

^b^ Five of the 22 patients who underwent salvage TL were initially treated with local radiotherapy for early stage laryngeal cancer. The remaining patients who underwent salvage TL were initially treated with locoregional radiotherapy for advanced stage laryngeal cancer. One of these 17 patients was treated with bioradiation (cetuximab; no. of patients = 1).

### 3.2 HADS and MDADI

Clinically relevant affective symptoms were seen in 11 (31%) patients. Eight (23%) patients scored 8 or more points on the HADS‐A subscale and 8 (23%) patients scored 8 or more points on the HADS‐D subscale. Five (14%) patients scored 8 or more points on both subscales. The mean (SD) HADS‐T score of all patients was 10.4 (7.9). The HADS subscale scores are shown in Table 2. Floor and ceiling effects were not found as none of the patients had the highest score of 21 and two (6%) patients scored the lowest score on the HADS‐A and HADS‐D subscales.

**TABLE 2:** The mean and standard deviation of the MDADI (sub)scale scores and the level of significance (*P*) for comparison (mean difference) between patients with vs without clinically relevant symptoms of anxiety and/or depression

MDADI/HADS subscale	Total	HADS‐A≥8	HADS‐A<8	Student's *t* test (*P*)	HADS‐D≥8	HADS‐D<8	Student's *t* test (*P*)	HADS‐A+D≥8	HADS‐A+D<8	
	No. of patients = 35 (100%)	No. of patients = 9 (24%)	No. of patients = 28 (76%)		No. of patients = 9 (24%)	No. of patients = 28 (76%)		No. of patients = 5 (14%)	No. of patients = 24 (65%)	
	Mean (SD)	Mean (SD)	Mean (SD)		Mean (SD)	Mean (SD)		Mean (SD)	Mean (SD)	Student's *t* test (*P*)
MDADI‐G	66 (29)	48 (15)	71 (30)	.006	50 (15)	70 (31)	.02	40 (0)	72 (32)	<.001
MDADI‐F	73 (2)	57 (15)	78 (19)	.006	57 (15)	78 (19)	.006	50 (14)	80 (20)	.004
MDADI‐P	70 (22)	48 (17)	76 (19)	.001	45 (20)	77 (17)	<.001	44 (15)	80 (15)	<.001
MDADI‐E	71 (18)	60 (21)	75 (16)	.04	53 (16)	77 (15)	<.001	48 (13)	76 (15)	.001
MDADI‐T	72 (19)	54 (15)	77 (16)	.001	51 (16)	78 (15)	<.001	47 (12)	79 (14)	<.001
HADS‐A	5.2 (4.3)	11.8 (3.3)	3.2 (2.0)	<.001	9.5 (5.5)	3.9 (2.9)	.02	12.8 (3.9)	3.1 (1.9)	.004
HADS‐D	5.1 (4.2)	9.9 (3.5)	3.7 (3.3)	<.001	11.5 (3.0)	3.3 (2.1)	<.001	11.8 (3.0)	2.9 (1.9)	<.001

Abbreviations: HADS‐A/D, Hospital Anxiety and Depression Scale ‐ Anxiety scale/Depression scale; MDADI‐G/F/P/E/T, MD Anderson Dysphagia Inventory ‐ Global/Functional/Emotional/Physical/Total; *P*, level of significance.

The median (IQR) MDADI‐T score was 68 (61‐87). The MDADI subscale scores are presented in Table 2. The MDADI questionnaire was reviewed for possible floor and ceiling effects as well. One (3%) respondent had the highest possible MDADI‐T score of 100 and none of the patients scored the lowest possible score of 20. The mean and SD of the MDADI (sub)scales and the level of significance (*P*) for comparison between patients with vs without clinically relevant symptoms of anxiety and/or depression are presented in Table 2. The mean scores of all MDADI subscales were significantly different between patients with vs patients without clinically relevant symptoms of anxiety, meaning swallow‐specific QoL seemed to be significantly lower in patients with anxiety symptoms. The same applies to all MDADI subscales when comparing patients with vs patients without clinically relevant symptoms of depression.

### 3.3 Prediction of MDADI scores based on affective symptom scores

**FIGURE 1:**
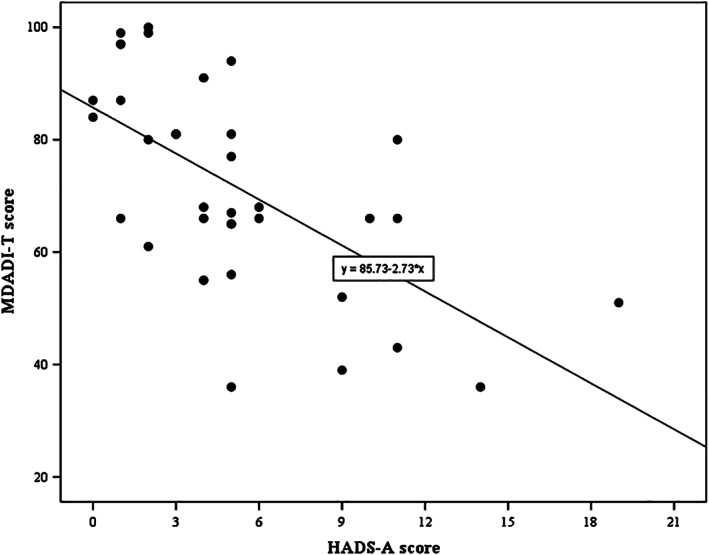
Linear regression analysis of MDADI‐T and HASDS‐A

**FIGURE 2:**
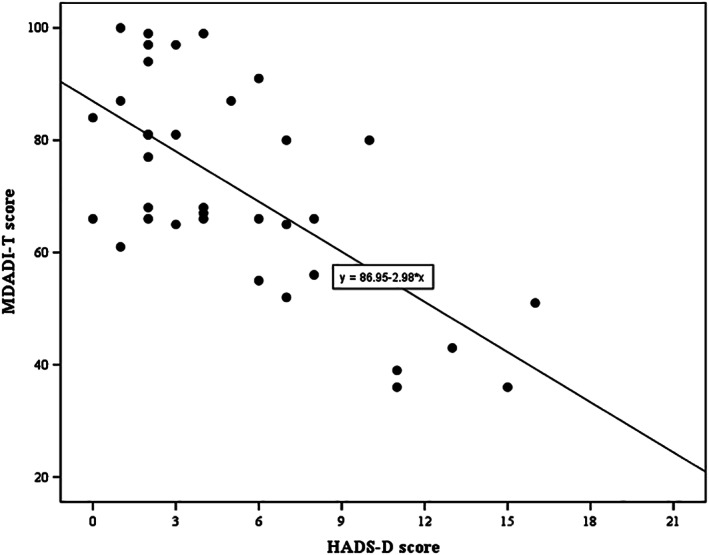
Linear regression analysis of MDADI‐T and HADS‐D

Patients' predicted MDADI‐T score was equal to 86‐2.7 (HADS‐A score), that is, the MDADI‐T score decreased, on average, 2.7 points for every point increase of the HADS‐A score (*P* < .001), representing a lower swallow‐specific QoL in TL patients who showed higher levels of anxiety. Differences in HADS‐A scores explained 38% of the variations in the MDADI‐T scores. Patients' predicted MDADI‐T score is equal to 87‐3.0 (HADS‐D score), that is, the MDADI‐T score decreased, on average, 3.0 for every point increase of the HADS‐D score (*P* < .001). Differences in HADS‐D scores explained 44% of the variations in the MDADI‐T scores. Linear regression analysis allows MDADI prediction using the HADS subscale scores. Regression slopes are between −3.7 and −2.1 for all MDADI subscales, meaning that one‐point increase of the HADS‐A or HADS‐D score corresponds with a decrease of 2.1 to 3.7 points of an MDADI subscale score, which indicates that increased anxiety or depression symptom scores correlate with a decreased swallow‐specific QoL. Results of each MDADI subscale are shown in Table 3. The prediction of MDADI‐T scores using HADS‐A and HADS‐D are displayed in Figures 1 and 2, respectively.

**TABLE 3:** Linear regression analysis of MDADI and HADS subscale scores. MDADI subscale scores are predicted using the HADS subscale scores in the linear regression equation

MDADI subscale	HADS‐A subscale			HADS‐D subscale		
	Adjusted *R* ^2^	Linear regression equation (SE)	*P*	Adjusted *R* ^2^	Linear regression equation (SE)	*P*
MDADI‐G	0.18	81.7 (7.0)‐3.1 (1.0)	.006	0.10	78.3 (7.4)‐2.4 (1.1)	.04
MDADI‐F	0.22	85.4 (4.8)‐2.4 (0.7)	.001	0.16	84.0 (5.0)‐2.1 (0.8)	<.009
MDADI‐P	0.38	86.8 (4.7)‐3.3 (0.7)	<.001	0.47	88.9 (4.3)‐3.7 (0.7)	<.001
MDADI‐E	0.26	82.6 (4.1)‐2.2 (0.6)	<.001	0.35	84.6 (3.9)‐2.6 (0.6)	<.001
MDADI‐T	0.38	85.7 (3.9)‐2.7 (0.6)	<.001	0.44	86.9 (3.7)‐3.0 (0.6)	<.001

Abbreviations: HADS‐A/D, Hospital Anxiety and Depression Scale ‐ Anxiety scale/Depression scale; MDADI‐G/F/P/E/T, MD Anderson Dysphagia Inventory ‐ Global/Functional/Emotional/Physical/Total; *P*, level of significance.

## 4 DISCUSSION

This study screened the prevalence of clinically relevant symptoms of anxiety and depression, and the level of swallow‐specific QoL in a population of TL patients with OD complaints. Furthermore, the relationship between affective symptoms and swallow‐specific QoL was explored. Clinically relevant symptoms of anxiety or depression were reported by approximately a third (31%) of the TL patients, and one in seven patients experienced both symptoms. A high prevalence of clinically relevant affective symptoms has been described previously in TL patients.^15^ Salturk et al used HADS‐A≥7 and HADS‐D≥5 as cutoff values for the confirmation of clinically relevant affective symptoms and identified that 60% of the TL patients presented clinically relevant symptoms of anxiety and 80% of depression.^1^ In another study, where cutoff values of ≥7 for both HADS‐A and HADS‐D were used, the authors reported that approximately one‐third of the TL patients experienced symptoms of either anxiety or depression.^15^ In the present study, a HADS‐A/D cutoff value ≥8 was used as this has been shown to provide an optimal balance between sensitivity and specificity.^23^ Different cutoff values affect the identification of TL patients with clinically relevant affective symptoms and preclude comparison between studies. Furthermore, the present study comprised only TL patients with OD, which may also have affected the prevalence of affective symptoms compared to non‐dysphagic TL patients. Currently, there is no consensus in the literature on what might be the optimal HADS‐A/D cutoff value in a TL population.^28^

The effect of different types of HNC treatment on the MDADI and HADS scores was considered in the statistical analysis. However, as stated earlier, these subgroups were considered too small for meaningful analysis. Radiotherapy is usually part of the multimodality treatment of TL patients and can lead to increased signs of OD due to xerostomia, pain, tissue swelling, fibrosis, lymphedema, or radiotherapy‐induced sensorial neuropathy in the long term.^8^ The majority of the patients in the present study underwent radiotherapy during the course of HNC treatment: as an adjuvant treatment whether or not with concurrent cisplatin following TL or as definitive (bio)radiotherapy before salvage TL.

Another interesting aspect of this study is the influence of the time interval between the end of treatment and the period of data collection. All HADS and MDADI questionnaires were completed at least 4 months after HNC treatment. It is possible that TL patients got used to the OD symptoms and adjusted to living with the limitations as time passed.^29^ The findings of the present study support this theory, as it was seen that the patients with clinically relevant affective symptoms completed the questionnaires significantly earlier following the end of treatment compared to patients without relevant symptoms. Another reason might be that this latter group of patients not only got used to OD but also to the TL itself, which has a profound impact on daily living. This hypothesis could not be tested, as data from nondysphagic TL patients was not available.

In the current study, the MDADI questionnaire was used to assess the level of swallow‐specific QoL. The median MDADI‐T score of 68 was similar to the one reported in a previous study on TL patients by Robertson et al.^30^ However, their study population comprised a TL sample without any information on the presence of OD.^30^

The relationship between health‐related QoL and affective symptoms in TL patients was examined earlier using different measurement tools. In the study by Maclean et al, 110 TL patients completed the World Health Organization QoL‐Bref (WHOQoL‐Bref) and the University of Washington QoL (UW‐QoL) questionnaire. Of these, the former does not include a domain about swallowing and the latter includes three questions on deglutition.^13^ The scores of these tools were compared with the scores of the Depression Anxiety and Stress Scale (DASS). Their results showed that OD may not necessarily affect the level of health‐related QoL following TL. However, dysphagic TL patients reported high levels of depression and anxiety symptom scores. The authors identified OD in these patients using patient self‐report information on any change in their swallowing ability or the need to change the texture of their diet after TL. In a study by Perry et al, the authors also examined TL patients using the WHOQoL‐Bref, the DASS questionnaire, and the Australian Therapy Outcome Measures (AusTOMs).^14^ Swallowing function in these patients was measured using the functioning domain of the AusTOMs and was shown to be a predictor for environmental QoL, although the authors concluded that symptoms of depression, anxiety, and stress had a higher impact on self‐perceived health‐related QoL than speech and swallowing function.

In this study, the presence of clinically relevant affective symptoms was accompanied by significantly lower scores of swallow‐specific QoL and one‐point increase in HADS‐A or HADS‐D scores correlated, on average, with 2.7 or 3.0 points decrease, respectively, in MDADI‐T score. The question remains whether TL patients can recognize these differences in daily life. The minimal clinically important difference (MCID) is the patient‐dependent difference in score due to a clinical intervention that reflects changes that are meaningful to the patient. The MCID could not be determined for the present cohort study due to its cross‐sectional design. Previously, it was determined that an MCID for the MDADI‐T scale in patients with HNC is 10 points, representing the smallest change in MDADI‐T outcome that would differentiate feeding tube dependent patients from non‐tube dependent ones and aspirators from non‐aspirators.^31^ An MCID was also calculated for the HADS, ranging between 1.3 and 1.8 for HADS‐A and between 1.5 and 1.7 for HADS‐D, although this was not confirmed in a HNC population.^32^, ^33^

Although a negative correlation between affective symptoms and swallow‐specific QoL scores was clearly seen, its causal direction could not be determined in this cross‐sectional study design. In other words, it is still unclear if affective symptoms can evoke or amplify OD resulting in lower swallow‐specific QoL scores or that OD and a lower swallow‐specific QoL may lead to higher depression and anxiety symptom scores. It is still unknown by what mechanisms OD is associated with affective symptoms, although several might be considered. Affective symptoms are known to cause physical complaints, such as a dry mouth,^34^ causing swallowing difficulty. Moreover, suffering from affective symptoms might subsequently hamper motivation during the HNC rehabilitation phase,^35^ which may result in poor functional outcome. A possible neurobiological explanation is that cerebral motor cortex areas relate to the neural stress connectome in case of affective symptoms, thereby affecting muscle control,^36^ which could certainly play a role in the motor control of swallowing in TL patients.

The authors of this study conclude that clinically relevant affective symptoms contribute to a lower swallow‐specific QoL or vice‐versa in dysphagic TL patients. As the HNC treatment outcome of TL patients comprises different measurable dimensions, such as purely physical functions, but also psychosocial functions, the overall follow‐up evaluation should address these multiple dimensions, too. Screening for underlying psychopathology and, in particular, affective disorders is usually done at the start of HNC treatment. After some time and certainly after completing the 5‐year follow‐up period, TL patients are in danger of getting out of sight. Even though affective symptoms seem to become less over time, we advocate easily accessible OD care for this patient group, including screening for affective disorders and swallow‐specific QoL alongside care for somatic illness.

### 4.1 Limitations of the study

This study has some limitations. First, since advanced stage larynx and hypopharynx cancer are low prevalent diseases, the number of included TL patients is small, so only limited statistical analysis without group stratification and adjustment in the regression model (for treatment modalities, psychotropic drugs, age, gender, time after treatment, etc.) could be performed. It is certainly possible that one or more of the aforementioned variables affected the HADS and/or MDADI score. Furthermore, a significant number of patients with advanced stage HNC die within 5 years after oncological treatment,^37^ making it again difficult to achieve a larger sample size. Second, the HADS questionnaire was used for the screening of clinically relevant affective symptoms, because it is one of the most frequently used and reliable questionnaires for this clinical issue. A different screening tool or multiple screening tools might have led to different results. Third, we do not know for sure whether there is a causal relationship between OD and the high prevalence of clinically relevant affective symptoms in TL patients. Other factors may play a role in this high prevalence too. Moreover, based on this cross‐sectional study design, we do not know the direction of a possible causal relationship between the aforementioned variables. Finally, during data extraction from the oncological patient files, it was noticed that data on family and employment status has only been thoroughly reported since the introduction of the Dutch Head and Neck Audit (a mandatory national database on head‐and‐neck cancer including standardized information on diagnostics, treatment, timeline, etc.). This means that the data from patients evaluated before 2018 contains a too high percentage of missing values on family and employment status preventing us to use this data for further analysis.^38^

## 5 CONCLUSION

Clinically relevant affective symptoms were present in a third of dysphagic TL patients. These preliminary results show that increased affective symptom scores correlate with a decreased swallow‐specific QoL. However, since we do not know the direction of the causal relationship between the two, it remains important to assess both dimensions as dysphagic TL patients will need a different approach in the OD treatment plan in case of deviating scores.

## CONFLICT OF INTEREST

The authors declared no potential conflicts of interest.

## References

[1] Salturk Z , Arslanoglu A , Ozdemir E , et al. How do voice restoration methods affect the psychological status of patients after total laryngectomy? HNO. 2016;64(3):163‐168. 26923487 10.1007/s00106-016-0134-x

[2] Noonan BJ , Hegarty J . The impact of total laryngectomy: the patient's perspective. Oncol Nurs Forum. 2010;37(3):293‐301. 20439213 10.1188/10.ONF.293-301

[3] Krishnan S , Maclean J . Practice of laryngectomy rehabilitation interventions: a perspective from Australia. Curr Opin Otolaryngol Head Neck Surg. 2013;21(3):224‐229. 23644734 10.1097/MOO.0b013e32836118aa

[4] Pignon JP , le Maitre A , Maillard E , Bourhis J . Meta‐analysis of chemotherapy in head and neck cancer (MACH‐NC): an update on 93 randomised trials and 17,346 patients. Radiother Oncol. 2009;92(1):4‐14. 19446902 10.1016/j.radonc.2009.04.014

[5] Hui Y , Wei WI , Yuen PW , Lam LK , Ho WK . Primary closure of pharyngeal remnant after total laryngectomy and partial pharyngectomy: how much residual mucosa is sufficient? Laryngoscope. 1996;106(4):490‐494. 8614227 10.1097/00005537-199604000-00018

[6] Maclean J , Szczesniak M , Cotton S , Cook I , Perry A . Impact of a laryngectomy and surgical closure technique on swallow biomechanics and dysphagia severity. Otolaryngol Head Neck Surg. 2011;144(1):21‐28. 21493382 10.1177/0194599810390906

[7] Maclean J , Cotton S , Perry A . Post‐laryngectomy: it's hard to swallow: an Australian study of prevalence and self‐reports of swallowing function after a total laryngectomy. Dysphagia. 2009;24(2):172‐179. 18784911 10.1007/s00455-008-9189-5

[8] Murphy BA , Gilbert J . Dysphagia in head and neck cancer patients treated with radiation: assessment, sequelae, and rehabilitation. Semin Radiat Oncol. 2009;19(1):35‐42. 19028344 10.1016/j.semradonc.2008.09.007

[9] Landera MA , Lundy DS , Sullivan PA . Dysphagia after total laryngectomy. Perspect Swal Swal Dis (Dysph). 2010;19(2):39‐44.

[10] Williams LR , Kasir D , Penny S , Homer JJ , Laasch HU . Radiological balloon dilatation of post‐treatment benign pharyngeal strictures. J Laryngol Otol. 2009;123(11):1229‐1232. 19607738 10.1017/S0022215109990508

[11] Gadepalli C , de Casso C , Silva S , Loughran S , Homer JJ . Functional results of pharyngo‐laryngectomy and total laryngectomy: a comparison. J Laryngol Otol. 2012;126(1):52‐57. 21867586 10.1017/S0022215111002313

[12] Terlingen LT , Pilz W , Kuijer M , Kremer B , Baijens LW . Diagnosis and treatment of oropharyngeal dysphagia after total laryngectomy with or without pharyngoesophageal reconstruction: systematic review. Head Neck. 2018;40(12):2733‐2748. 30478930 10.1002/hed.25508 PMC6587738

[13] Maclean J , Cotton S , Perry A . Dysphagia following a total laryngectomy: the effect on quality of life, functioning, and psychological well‐being. Dysphagia. 2009;24(3):314‐321. 19290578 10.1007/s00455-009-9209-0

[14] Perry A , Casey E , Cotton S . Quality of life after total laryngectomy: functioning, psychological well‐being and self‐efficacy. Int J Lang Commun Disord. 2015;50(4):467‐475. 25703153 10.1111/1460-6984.12148

[15] Danker H , Wollbruck D , Singer S , Fuchs M , Brahler E , Meyer A . Social withdrawal after laryngectomy. Eur Arch Otorhinolaryngol. 2010;267(4):593‐600. 19760214 10.1007/s00405-009-1087-4

[16] Vilaseca I , Chen AY , Backscheider AG . Long‐term quality of life after total laryngectomy. Head Neck. 2006;28(4):313‐320. 16200627 10.1002/hed.20268

[17] Blanco‐Pinero N , Antequera‐Jurado R , Rodriguez‐Franco L , Ibanez‐Guerra E , Herrero‐Salado TF , Sanchez‐Gomez S . Emotional and psychopathological disorders in laryngectomized oncological patients. Acta Otorrinolaringol Esp. 2015;66(4):210‐217. 25465282 10.1016/j.otorri.2014.09.006

[18] Braz DS , Ribas MM , Dedivitis RA , Nishimoto IN , Barros AP . Quality of life and depression in patients undergoing total and partial laryngectomy. Clinics (Sao Paulo, Brazil). 2005;60(2):135‐142. 10.1590/s1807-59322005000200010 15880250

[19] Singer S , Danker H , Bloching M , et al. Perceived stigmatisation following laryngectomy. Psychother Psychosom Med Psychol. 2007;57(8):328‐333. 17334971 10.1055/s-2006-952016

[20] Union for International Cancer Control . TNM Classification of Malignant Tumours. 7th ed. Hoboken, NJ: Wiley‐Blackwell; 2009.

[21] CCMO . Non‐WMO research. https://english.ccmo.nl/investigators/types-of-research/non-wmo-research

[22] Crary MA , Mann GD , Groher ME . Initial psychometric assessment of a functional oral intake scale for dysphagia in stroke patients. Arch Phys Med Rehabil. 2005;86(8):1516‐1520. 16084801 10.1016/j.apmr.2004.11.049

[23] Bjelland I , Dahl AA , Haug TT , Neckelmann D . The validity of the hospital anxiety and depression scale. An updated literature review. J Psychosom Res. 2002;52(2):69‐77. 11832252 10.1016/s0022-3999(01)00296-3

[24] Zigmond AS , Snaith RP . The hospital anxiety and depression scale. Acta Psychiatr Scand. 1983;67(6):361‐370. 6880820 10.1111/j.1600-0447.1983.tb09716.x

[25] Chen AY , Frankowski R , Bishop‐Leone J , et al. The development and validation of a dysphagia‐specific quality‐of‐life questionnaire for patients with head and neck cancer: the M. D. Anderson dysphagia inventory. Arch Otolaryngol Head Neck Surg. 2001;127(7):870‐876. 11448365

[26] Spinhoven P , Ormel J , Sloekers PP , Kempen GI , Speckens AE , Van Hemert AM . A validation study of the hospital anxiety and depression scale (HADS) in different groups of Dutch subjects. Psychol Med. 1997;27(2):363‐370. 9089829 10.1017/s0033291796004382

[27] Speyer R , Heijnen BJ , Baijens LW , et al. Quality of life in oncological patients with oropharyngeal dysphagia: validity and reliability of the Dutch version of the MD Anderson Dysphagia Inventory and the Deglutition Handicap Index. Dysphagia. 2011;26(4):407‐414. 21279522 10.1007/s00455-011-9327-3 PMC3224721

[28] Mitchell AJ , Meader N , Symonds P . Diagnostic validity of the Hospital Anxiety and Depression Scale (HADS) in cancer and palliative settings: a meta‐analysis. J Affect Disord. 2010;126(3):335‐348. 20207007 10.1016/j.jad.2010.01.067

[29] Hammerlid E , Taft C . Health‐related quality of life in long‐term head and neck cancer survivors: a comparison with general population norms. Br J Cancer. 2001;84(2):149‐156. 11161369 10.1054/bjoc.2000.1576 PMC2363699

[30] Robertson SM , Yeo JC , Dunnet C , Young D , Mackenzie K . Voice, swallowing, and quality of life after total laryngectomy: results of the west of Scotland laryngectomy audit. Head Neck. 2012;34(1):59‐65. 21416548 10.1002/hed.21692

[31] Hutcheson KA , Barrow MP , Lisec A , Barringer DA , Gries K , Lewin JS . What is a clinically relevant difference in MDADI scores between groups of head and neck cancer patients? Laryngoscope. 2016;126(5):1108‐1113. 26542529 10.1002/lary.25778 PMC4842113

[32] Smid DE , Franssen FM , Houben‐Wilke S , et al. Responsiveness and MCID estimates for CAT, CCQ, and HADS in patients with COPD undergoing pulmonary rehabilitation: a prospective analysis. J Am Med Dir Assoc. 2017;18(1):53‐58. 27624705 10.1016/j.jamda.2016.08.002

[33] Lemay KR , Tulloch HE , Pipe AL , Reed JL . Establishing the minimal clinically important difference for the hospital anxiety and depression scale in patients with cardiovascular disease. J Cardiopulm Rehabil Prev. 2019;39(6):E6–E11. 30489438 10.1097/HCR.0000000000000379

[34] Gholami N , Hosseini Sabzvari B , Razzaghi A , Salah S . Effect of stress, anxiety and depression on unstimulated salivary flow rate and xerostomia. J Dent Res Dent Clin Dent Prospects. 2017;11(4):247‐252. 29354252 10.15171/joddd.2017.043 PMC5768958

[35] Dickson JM , Johnson S , Huntley CD , Peckham A , Taylor PJ . An integrative study of motivation and goal regulation processes in subclinical anxiety, depression and hypomania. Psychiatry Res. 2017;256:6‐12. 28618249 10.1016/j.psychres.2017.06.002

[36] Verdonschot R , Baijens LWJ , Vanbelle S , van de Kolk I , Kremer B , Leue C . Affective symptoms in patients with oropharyngeal dysphagia: a systematic review. J Psychosom Res. 2017;97:102‐110. 28606489 10.1016/j.jpsychores.2017.04.006

[37] Jehn P , Dittmann J , Zimmerer R , et al. Survival rates according to tumour location in patients with surgically treated oral and oropharyngeal squamous cell carcinoma. Anticancer Res. 2019;39(5):2527‐2533. 31092449 10.21873/anticanres.13374

[38] Van Overveld L . Quality of Care in Head and Neck Oncology. Nijmegen, the Netherlands: IQ Healthcare, Radboud University; 2018.

